# A Protest against the Fear of Dermatitis Originating from Exposure to the Roentgen Rays*Read before the Denver and Arapahoe Medical Society, February 25, 1898.

**Published:** 1898-04

**Authors:** G. H. Stover

**Affiliations:** Assistant in Medicine in the University of Denver


### A PROTEST AGAINST THE FEAR OF DERMATITIS ORIGINATING FROM EXPOSURE TO THE ROENTGEN RAYS.*

BY G. H. STOVER, M.D.

Assistant in Medicine in the University of Denver.

There is gaining ground in the minds of the laity at the present time in this city an unfortunate fear of burns from the X-ray. It has grown to such an extent as to seriously hamper some of our surgeons who appreciate the value of the X-ray in diagnosis and treatment. I attribute this fear to two principal causes; one is the fact of a publication some time ago in a sensational New York daily of the case of a young lady, one side of whose head was burned to such an extent that the hair on that side was lost. This paper gave a lurid description of the injury, with the usual horrible illustrations, and the story was widely copied by the press of the whole country. The other cause of fear in Denver was the reported severe burning of the hands of a local X-ray operator. The affair was given wide publicity by the city press, articles appearing for some days.

The present course of this local electrician is calculated to alarm still more the already timid laity. I understand that the prospective subject of X-ray investigation is required to sign a waiver of damages, on account of injury from the X-ray. These elaborate precautions can not fail to impress upon the patient the notion that the X-ray is a very dangerous agent. Of course, there is some argument in favor of such precautions if the operator is timid, unskillful, or wealthy; in my own case, the first and last conditions at least, are not found, so I do not believe such precautions necessary. And any way, in the present state of nearly perfect apparatus, I think the physician who gives a dose of calomel might almost as reasonably exact a release from the patient, for the reason that certain unfortunates have lost their teeth as a result of salivation. I have tried to see the Denver operator who was said to have been burned, but have been unable to do so; I have heard from various parties who claim to know him, various reports; one said that one hand was burned; another that both hands suffered; another added a severe burn of the chest; my last informant said it had been a case of German measles!

At this time I feel like saying a few words to the members of this society, to tell them that the X-ray is not at all the deadly weapon that some suppose it to be; that with an X-ray generated by proper apparatus and applied by a competent operator, there is no danger of burns. In making this

*Read before the Denver and Arapahoe Medical Society, February 25, 1898.

statement I wish it distinctly understood that I cast *no* reflection upon the skill of the operator whom I have mentioned; that would be absurd. He is known to you all as an electrician of ability; some of the pictures he has produced are as fine as have been produced in the country, and he deserves great credit for his enterprise, manifested at a very early period in X-ray history, and his courtesies to the medical profession of the city and State will long be remembered. A great deal of his work was done without any thought of monetary compensation.

My claim is that all the burns that have followed the use of the X-ray are the result of:

1. Improper apparatus.
2. Too long exposures.
3. Too small a distance between the tube and the object being skiagraphed.

As to the first, I want to say that the induction coil is not a proper agent for the generation of the current that is applied to the tube. The static machine is what should be used. The static, Franklinic, or influence machine, as it is called, will, when of a proper size, give just as good a fluoroscopic image, just as good a skiägraph as the most efficient induction coil, and without the disadvantages of the latter; the Franklinic machine never heats the tube over blood heat, and usually the tube remains *cool*. It is not necessary with the static machine to stop operation and cool the tube; neither does one have to keep a fan blowing upon it to keep it cool. The static current never causes the tube to become red hot and puncture at the point of exit of the rays. The snapping of the spark, so disagreeable in the coil, is obviated by the influence machine. A good tube may be kept in operation for hours without deterioration of the ray. There is never any danger of "burning out" a static machine, thus ruining the whole apparatus, as is the danger with a street current and coil.

I have never known of a case of dermatitis following the use of the Franklinically generated X-ray when used in a rational manner. I am informed by Prof. S. H. Monell of Brooklyn that the static machine has produced six or eight burns, but that a disrputive discharge was used. This is not a proper current.

As regards the second and third factors in the production of X-ray burns, it should be said that with the highly efficient tubes we may now procure, there is no need to place the object of investigation in dangerous proximity to the tube, nor are the hour and a-half exposures any longer necessary. The object should not be placed too close to the tube, as the image is thereby distorted, which I shall show more fully in a future paper.

The chief factor in the causation of X-ray burns, however, lies in the kind of current applied to the tube. This should properly be generated by a powerful Franklinic machine, and the *convective* discharge should be used.

Prof. Monell, who is a leader in this line, and other investigators have an experience similar to mine in the use of the Franklinic machine for X-ray work.

Every day, for a longer or shorter time, I am bathed in the X-rays. Probably thirty times I have stood for fifteen minutes at a time, with my chest a couple of inches from the tube, while others were looking at my heart and other internal structures through the fluoroscope. My arms and hands have been examined by fluoroscope and skiagraph a great many times. The hip, foot and knee have also been frequently subjected to the X-ray. I frequently place my face within a few inches of the tube, so that others may view with the fluoroscope the weird picture of a living skull, and I have not experienced the slightest ill effects. I use a Waite & Bartlett eight-plate influence machine, convective discharge, and at present a Monell tube with twelve inches between external electrodes, exhausted to one one-millionth of an atmosphere.

I cite these experiences of my own simply to show that I believe it, when I say that there need be no danger of X-ray burns.

As to the burns which have occurred, I believe them to have been directly or indirectly the result chiefly of too heavy electrical discharges in operating the tube.

As little is known as to the exact cause and nature of the so-called burns as is known of the nature of the X-ray itself. It must be a penetrating agent, for there are reported, and I have seen, neuritis and periostitis, as well as dermatitis in these cases. It is probably not a burn; if it is, it is not caused by the X-ray itself, which has no calorific property. It is probably not due to the action of particles of platinum from the electrodes, for delicate chemical analysis of the injured tissues has failed to show a trace of platinum. It is proven that the injury is not caused by ultra violet light rays. It has been suggested that the cathode ray may be the mischief. maker. As I said before, I think the chief factor lies in the kind of current used.

My points may be summed up in these words: With a proper current-generating apparatus, a tube of good efficiency, and the rays applied in a proper manner, there is no danger of a burn, or whatever these injuries are.

I hope that what I have said in this elementary talk may be of use to you in overcoming this mistaken fear.—*Western Medical and Surgical Gazette.*

